# Book Reviews

**Published:** 1912-01

**Authors:** 


### BOOK OF VIEWS.

---

ANATOMY. A Manual for Students and Practitioners. By John F. Little, M. D, of the JeJfferson Medical College, Philadelphia. New (2nd) edition, enlarged and thoroughly revised. 12mo, 491 pages, with 75 engravings. Double number. Cloth $1.50 net. The Medical Epitome Series. Lea & Febiger, Publishers, Philadelphia and New York, 1911.

That this work has fulfilled the purpose for which it was designed is shown by the demand, which has exhausted several printings of the first edition, and has now led to the call for a revision, in which it has been brought thoroughly up to date and improved in many ways. The present revision has been placed in the hands of Dr. John F. Little, of the Jefferson Medical College, Philadelphia. This arrangement is an admirable one, for not only is the author well equipped for his task, but he has also availed himself of the valuable suggestions of Professor E. A. Spitzka, one of the foremost anatomists of this country. The volume therefore should be of the utmost assistance to students for purposes of quizzing, and to physicians and surgeons for

---

ELECTRICITY, ITS MEDICAL AND SURGICAL APPLICATIONS, INCLUDING RADIOTHERAPY AND PHOTOTHERAPY. By Charles S. Potts, M. D., Professor of Neurology in the Medico-Chirurgical College of Philadelphia, with a Section on Electrophysics by H. C. Richards, Ph. D., and a Section on X-rays by H. K. Pancoast, M. D., of the University of Pennsylvania. Octavo, 509 pages, with 356 illustrations and 6 plates. Cloth, $4.75 net. Lea & Febiger, Publishers, Philadelphia and New York, 1911.

The books opens with a section on the physics of electricity, from the pen of Dr. Horace Clark Richards, Professor of Mathematical Physics in the University of Pennsylvania. Dr. Potts then follow with sections on electrophysiology, electrodiagnosis and prognosis, general electrotherapeutics, methods of obtaining general and local effects by indirect action of electricity, such as the Finsen light, and special electrotherapeutics. The volume closes with a section on the application of the X-rays, written by Dr. H. K. Pancoast, Professor of Roentgenology in the University cf Pennsylvania, than whom no specialist on the subject stands higher. The text is elaborately illustrated. The work is moderate in size, clear in its presentations, comprehensive, modern, and suitable alike for the physican and surgeon, as well as for the student.

---

A TEXT-BOOK OF MEDICAL CHEMISTRY AND TOXICOLOGY. By James W. Holland, M. D., Professor of Medical Chemistry and Toxicology, Jefferson Medical College, Philadelphia. Third Revised Edition. Octave of 655 pages, fully illustrated. Phiadelphia and London: W. B. Saunders Company, 1911. Cloth, $3.00 net.

Holland has succeeded well in presenting clearly and with due proportion medical chemistry and toxicology, with the avoidance of extraneous matters which only complicate the study for the student and practitioner. This third edition has been brought thoroughly up to date.

---

INFECTIONS OF THE HAND. A GUIDE TO THE SURGICAL TREATMENT OF ACUTE AND CHRONIC SUPPURATIVE PROCESSES IN THE FINGERS, HAND AND FOREARM. By Allen B. Kanavel, M. D., Assistant Professor of Surgery, Northwestern University Medical School, Chicago. Octavo, 447 pages, with 133 illustrations. Cloth, $3.75 net. Lea & Febiger, Philadelphia and New York, 1912.

Anatomically the hand is tremendously compact, particularly n its various muscles, tendons and fasciae, and its direct connection with the lymphatics of the arm makes its infections highly dangerous. Dr. Kanavel has made a special study of all these problems, and has been able to reduce the hitherto unsatisfactoy surgery of the hand to comparatively simple principles and direcions. He has illustrated his work with many original drawings. Such a work will be needed not only by every surgeon, but also by all general practitioners, for injuries of the hand are met with everywhere, and require prompt and proper care. It is rare that any medical book can be said to ender a high public service, but any medical book can be said to render a high public service, but

